# A parallel dual-stream state-space module for reliable and efficient biomedical relation extraction

**DOI:** 10.1371/journal.pcbi.1014480

**Published:** 2026-07-30

**Authors:** Yaxun Jia, Zhu Yuan, Lian Zhu, Bing Han, Li Ren, Zuo-lin Xiang

**Affiliations:** 1 Department of Radiation Oncology, Shanghai East Hospital, Tongji University School of Medicine, Shanghai, China; 2 Department of Information Management, The National Police University for Criminal Justice, Baoding, China; Xinjiang Technical Institute of Physics and Chemistry, CHINA

## Abstract

Automated drug-drug interaction (DDI) extraction is a cornerstone of global pharmacovigilance, yet its progress is stymied by a fundamental linguistic paradox: relations are signaled by localized morphological cues while being governed by long-range semantic logic. Current monolithic architectures, including Transformer-based models, often face challenges in resolving this feature entanglement, where local clinical descriptors often distort the distal logical chain, leading to noise propagation and reasoning failures. To address this, we present DuSSM, a parallel state-space framework that structurally disentangles surface patterns from semantic evolution. DuSSM implements a bifurcated pipeline: an explicit convolutional stream acting as a local pattern recognizer to isolate syntactic triggers, and an implicit stream leveraging selective state-space modeling (Mamba) to maintain stable semantic states. Although the initial contextual encoding retains a quadratic complexity ($𝒪(N^{2})$), this decoupled downstream reasoning module operates with a strictly linear 𝒪(N) complexity. Our extensive experiments across four diverse biomedical benchmarks (DDI-2013, ChemProt, GAD, EU-ADR) demonstrate that this dual-stream feature separation generalizes exceptionally well, achieving a robust Fl-score of 82.27% on the DDI benchmark. Notably, DuSSM yields 94.32% precision on non-interaction cases, effectively mitigating the alert fatigue that can arise from the probabilistic nature of generative large language models (LLMs) in zero-shot settings. By reconciling computational efficiency with mechanistic interpretability, DuSSM provides a scalable and trustworthy paradigm for deciphering complex biological interactions within massive electronic health records. All code and data have been publicly released at: https://github.com/Hero-Legend/DuSSM.

## Author summary

Automated systems that read medical records to find dangerous drug-drug interactions are crucial for patient safety. However, these systems often struggle to process complex medical language, frequently confusing localized cue words with the broader, long-distance logical context of a sentence. This confusion leads to a high number of false alarms, which can overwhelm clinicians. To solve this, we developed a new computational framework called DuSSM. Instead of processing clinical texts as a single entangled stream, our reasoning module uses two separate pathways: one specifically isolates distinct morphological keywords, while the other independently tracks the underlying logical trajectory across the entire sentence. By keeping these two processes decoupled, our system prevents local syntactic noise from distorting global reasoning. When tested across multiple diverse biomedical datasets—encompassing drug, chemical, and genetic interactions—our approach not only maintained high sensitivity but also significantly reduced false positive alerts. Furthermore, our architecture operates much faster and requires less computational memory than current standard models. Ultimately, our framework provides a more reliable, efficient, and transparent tool for hospitals to automatically screen massive electronic health records, helping to ensure medication safety without burdening doctors with unnecessary warnings.

## Introduction

**Fig 1:**
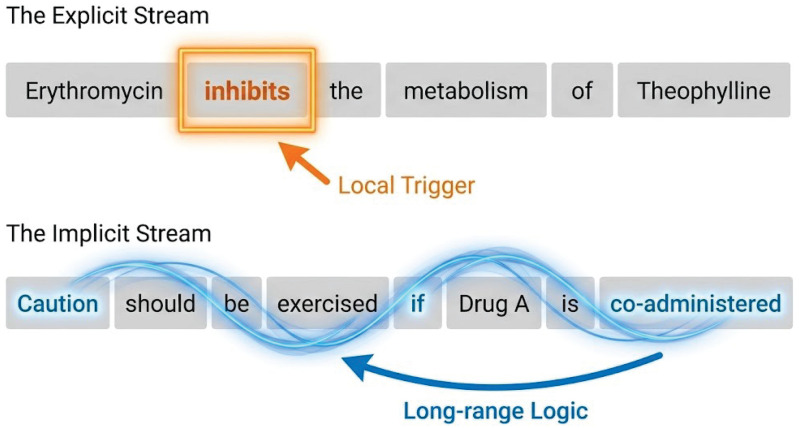
The challenge of linguistic duality in DDI extraction.

The rapid expansion of pharmacological knowledge and the increasing complexity of multi-drug regimens have elevated automated drug-drug interaction (DDI) extraction to a critical pillar of modern pharmacovigilance [1–5]. Concurrently, the broader landscape of biomedical informatics has been significantly revolutionized by advanced deep learning and graph representation methodologies. For instance, sophisticated sequence-based deep learning models have been successfully deployed for complex biological sequence predictions, such as identifying HIV-1 protease cleavage sites [6], while multi-view contrastive learning frameworks have pushed the boundaries of DDI event prediction over complex pharmacological graphs [7]. These contemporary innovations clearly highlight the immense potential of advanced neural architectures in capturing intricate biochemical dependencies. However, translating this advanced predictive power to unstructured clinical narratives presents a unique challenge. At its core, the extraction of biochemical relations from unstructured clinical narratives is not merely a pattern-matching task but a complex linguistic decoding process. A fundamental scientific challenge arises from the linguistic duality of medical text: interactions are often signaled by localized, high-frequency morphological triggers (e.g., “inhibition,” “concomitant”) while simultaneously being governed by implicit semantic logic that spans extensive, multi-clause dependencies [8–10] (Fig 1). Capturing this interplay is essential for the reliability of automated systems in real-world clinical settings.

Recent advancements in biomedical NLP have been dominated by Transformer-based architectures, which rely on global self-attention mechanisms to model dependencies [11–19]. However, these monolithic serial models can face challenges regarding feature entanglement. By processing local syntactic cues and global semantic states within the same attention space, they are prone to noise propagation, where localized clinical descriptions can distort the long-range logical chain. Furthermore, the quadratic computational complexity ($𝒪(N^{2})$) of Transformers imposes a scalability ceiling, rendering them inefficient for the high-throughput screening of long-form electronic health records (EHRs). While large language models (LLMs) have emerged as powerful alternatives with remarkable zero-shot capabilities, their generative nature can occasionally lead to misinterpretations of complex negations or hypothetical contexts, presenting unique challenges for strict, deterministic clinical applications [20–22].

To resolve these limitations, we propose DuSSM (Dual-Stream State-Space Model), a framework designed to disentangle explicit patterns from implicit logic. Unlike previous methods that fuse features sequentially, DuSSM introduces a parallel dual-view architecture. The framework comprises two orthogonal streams: (1) An explicit stream utilizing a local 1D-CNN that functions as a morphological pattern recognizer to capture high-frequency syntactic triggers; and (2) An implicit stream leveraging the Mamba architecture, a selective state-space model (SSM) with linear complexity, acting as a logical reasoner. The Mamba module maintains a continuous memory state that evolves over the sequence, allowing the model to capture subtle, long-range causal dependencies without the computational overhead of self-attention. By fusing these disentangled views, DuSSM calibrates the decision boundary to ensure that interactions are predicted only when syntactic evidence aligns with logical validity.

The key contributions of this study are summarized as follows:

- We propose DuSSM, a parallel reasoning framework that structurally disentangles morphological feature extraction from topological logic. This dual-stream design addresses the feature entanglement challenges often observed in traditional serial stacking architectures.
- We introduce a selective state-space formulation for relation extraction, modeling biomedical text as a continuous dynamic system. This approach effectively captures implicit logical dependencies across long spans.
- Extensive experiments across four diverse biomedical corpora (DDI-2013, ChemProt, GAD, and EU-ADR) demonstrate that DuSSM successfully addresses the critical challenge of false positives in pharmacovigilance. It maintains a competitive Macro F1-score of 82.27% on DDI-2013, ensuring high trustworthiness for real-world deployment.
- By leveraging the linear computational complexity (𝒪(N)) of the Mamba module, the downstream reasoning framework overcomes the quadratic bottleneck of traditional attention mechanisms typically used for feature fusion. Although the initial contextual encoder remains $𝒪(N^{2})$, our hybrid architecture still achieves an inference speedup of 1.6x over BioBERT baselines, facilitating the scalable processing of massive biomedical corpora.

## Related work

The extraction of drug-drug interactions (DDIs) has evolved from early rule-based systems to sophisticated deep learning architectures. Deep learning currently represents the dominant paradigm, broadly categorized by the granularity of feature extraction and the underlying mechanism for sequence modeling.

### Deep learning approaches

Early neural approaches predominantly utilized convolutional neural networks (CNNs) to capture local syntactic triggers, as demonstrated by models like DCNN [23] and SCNN [24]. While highly efficient at extracting explicit morphological cues within localized windows, CNNs inherently struggle to capture long-range dependencies across complex sentence structures. To bridge this gap, recurrent neural networks (RNNs) [25–27] and graph neural networks (GNNs) [28,29] were introduced to model sequential continuity and dependency trees. More recently, Transformer-based models (e.g., BioBERT [30], MEAT-BioBERT [31]) have established state-of-the-art results by leveraging global self-attention mechanisms for deep semantic modeling. Advanced variants have further enhanced performance by incorporating external knowledge graphs or multi-task learning strategies [32–35].

However, single-paradigm architectures often face an inherent trade-off: CNNs may miss the global context required for logical inference, while Transformers, despite their global receptive field, typically suffer from a quadratic computational complexity ($𝒪(N^{2})$) and can overlook fine-grained local syntactic triggers due to dispersed attention weights.

### State-space models and hybrid frameworks

State-space models (SSMs), particularly the recently proposed Mamba architecture [36], have emerged as a powerful alternative for long-sequence modeling. Unlike Transformers, which calculate pairwise attention scores, Mamba compresses historical context into a continuous latent state, enabling highly efficient linear-time propagation (𝒪(N)) [37,38]. This mathematical property makes Mamba exceptionally suitable for modeling the implicit logical dynamics that span long distances in biomedical narratives.

Despite its success in general domains, the application of selective SSMs in biomedical relation extraction remains underexplored. While Mamba excels at modeling sequential continuity (implicit logic), it lacks the explicit token-to-token alignment capability of attention mechanisms or the sharp focus of CNNs on localized triggers. Consequently, current approaches often suffer from a single-granularity bias, focusing excessively on either local syntax or global semantics.

To address these limitations, we propose DuSSM as a parallel reasoning framework. Unlike previous hybrid models that rely on sequential layer stacking, DuSSM utilizes a parallel architecture to structurally disentangle feature spaces. It assigns the morphological pattern recognition task to an explicit stream (powered by a local 1D-CNN) and delegates the long-range logical reasoning task to an implicit stream (driven by Mamba). This structural synergy allows the reasoning module to simultaneously capture high-frequency triggers and subtle semantic evolution, ensuring robust determinism and high precision even on severely imbalanced datasets.

## Materials and methods

**Fig 2:**
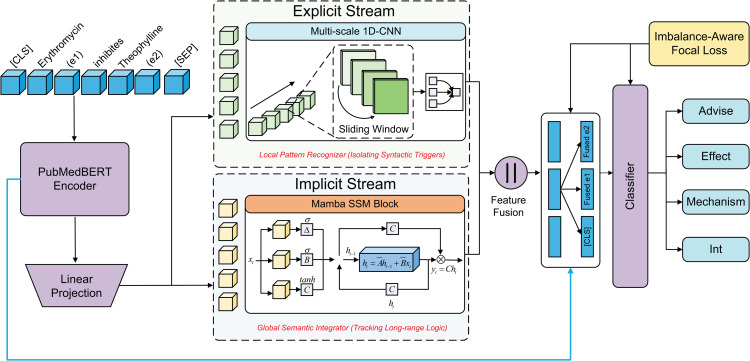
Overview of the proposed DuSSM architecture.

In this section, we formalize the mathematical framework of DuSSM. Grounded in the principle of linguistic disentanglement, DuSSM is designed to decompose complex biomedical narratives through two parallel pathways: isolating local keyword triggers from long-range semantic logic. The conceptual and structural workflow is summarized in Fig 2.

### Dual-stream manifold decomposition

As illustrated in Fig 2, DuSSM operates on the hypothesis that biomedical relations exhibit a signal duality: high-frequency explicit triggers ${ℋ}_{exp}$ (e.g., enzymatic verbs) and low-frequency implicit logic ${ℋ}_{imp}$ (e.g., causal trajectories across long distances). To prevent the mutual interference of these features, we formalize the overall feature space ℋ as the direct sum of two orthogonal subspaces:


$$\begin{array}{c} ℋ=\Phi_{exp}(ℋ)⊕\Phi_{imp}(ℋ) \end{array}$$ (1)

where $\Phi_{exp}$ acts as a local pattern recognizer to capture specific syntactic triggers, and $\Phi_{imp}$ serves as a global semantic integrator. We implement this via a four-stage pipeline: (1) Contextual encoding: Mapping tokens to a high-dimensional manifold using a PubMedBERT backbone; (2) Dual-stream feature separation: Projecting embeddings into two specialized subspaces; (3) Entity-aware fusion: Calibrating semantic features at precise entity coordinates; (4) Imbalance-aware optimization: Refining the decision boundary via an adaptive focal manifold.

Crucially, the necessity of a parallel dual-stream design, as opposed to conventional sequential hybrid models, stems from the problem of feature interference. In a standard sequential architecture, if an explicit local trigger (captured by the CNN) is fed serially into a state-space module, its sharp, high-frequency signal is inevitably smoothed out or diluted by the continuous recurrent state transitions of the global sequence. Conversely, if Mamba precedes the CNN, the global semantic logic is fragmented by the local pooling operations.

To mathematically prevent this interference, DuSSM introduces a strictly parallel topological design. By processing the projected space $H_{proj}$ through two independent, orthogonal pathways, the Explicit Stream acts as an uncorrupted high-pass filter for local triggers, while the Implicit Stream independently models the global semantic trajectory without being biased by localized noise. This structural disentanglement ensures that both morphological patterns and long-range logic are preserved in their native feature manifolds before being robustly integrated at the final entity-aware fusion stage.

### Input representation and feature projection

Let $S=\{w_{1},w_{2},...,w_{n}\}$ be an input sequence containing target entities *e*_1_ and *e*_2_. We leverage PubMedBERT [39], pre-trained on 14 million PubMed abstracts, to ensure the latent representation is natively aligned with the clinical domain. In our implementation, we freeze the lower layers of the encoder to retain general syntactic knowledge while fine-tuning the upper layers to adapt to the relational extraction task.

It is important to acknowledge that the contextual encoding phase relies on a Transformer backbone, which theoretically possesses a quadratic $𝒪(N^{2})$ computational complexity and a hard maximum sequence limit of 512 tokens. However, clinical sentences in standard DDI corpora are typically short (e.g., average length ≈ 42 tokens, truncated at a maximum of 300 tokens in our preprocessing). Within this practical length distribution, the quadratic overhead of the encoder is mathematically and empirically negligible. The true computational bottleneck and logical entanglement in modern biomedical extraction typically occur in the subsequent relational reasoning and feature fusion phases. Therefore, our core architectural contribution lies in replacing traditional computationally heavy cross-attention mechanisms with the proposed linear-time 𝒪(N) DuSSM reasoning module.

To bridge the contextual encoder and the reasoning module, the contextual embeddings $H_{enc}$ are subsequently projected into a shared lower-dimensional latent space via a linear transformation, yielding $H_{proj}$:


$$\begin{array}{c} H_{proj}=\text{GELU}(W_{p}H_{enc}+b_{p}) \end{array}$$ (2)

### Dual-stream disentanglement module

To resolve the entanglement inherent in monolithic serial models, we design two parallel pathways that process $H_{proj}$ independently.

#### Explicit stream: Local pattern recognizer.

The explicit stream is engineered as a local 1D-CNN to capture lexical spikes with high sensitivity. From a signal processing perspective, the local convolution functions as a high-pass filter that isolates sparse, high-energy morphological cues. For a kernel window *K*, the response at position *t* is formulated as:


$$\begin{array}{c} h_{cnn}^{\left(t\right)}=\text{GELU}\left(\sum_{k=1}^{K}W_{cnn}^{\left(k\right)}\cdot H_{proj}^{\left(t+k-\lfloor K/2\rfloor \right)}\right) \end{array}$$ (3)

This mechanism ensures that precise triggers are preserved while background semantic noise is effectively attenuated.

#### Implicit stream: Selective state-space dynamics.

In parallel, the implicit stream models the global semantic trajectory using a selective state-space model (Mamba). Mamba maps the projected input token $x_{t}\in H_{proj}$ to a latent state $h_{t}$ via discretized dynamics:


$$\begin{array}{c} h_{t}=\bar{𝐀}h_{t-1}+{\bar{𝐁}}_{t}x_{t},\;y_{t}={𝐂}_{t}h_{t} \end{array}$$ (4)

where $\bar{𝐀}=\exp(\Delta_{t}𝐀)$. Crucially, the model achieves logical selectivity through data-dependent parameters:


$$\begin{array}{cc} {𝐁}_{t} & ={\text{Linear}}_{B}(x_{t}),\\ {𝐂}_{t} & ={\text{Linear}}_{C}(x_{t}),\\ \Delta_{t} & =\text{Softplus}({\text{Linear}}_{\Delta }(x_{t})) \end{array}$$ (5)

This formulation allows for dynamic semantic filtering: the step size $\Delta_{t}$ adaptively contracts to skip parenthetical clinical background and expands to record critical logical evolution. This ensures a strict 𝒪(N) complexity while maintaining a stable semantic memory orthogonal to the explicit stream’s local responses.

#### Feature fusion and orthogonal calibration.

To establish a robust decision boundary, we integrate the disentangled views at entity coordinates $p_{e1}$ and $p_{e2}$. The fused representation $H_{fused}$ ensures that localized triggers do not overshadow long-range logical constraints. We achieve this by concatenating the independent features along the channel dimension:


$$\begin{array}{c} H_{fused}=[X_{cnn}∥X_{mamba}]\in {\mathbb{R}}^{L\times 2d_{model}} \end{array}$$ (6)

The final classification vector $v_{final}$ integrates the global representation with the target-specific disentangled semantics via concatenation:


$$\begin{array}{c} v_{final}=[H_{enc}^{\left[CLS\right]}∥H_{fused}^{\left(p_{e1}\right)}∥H_{fused}^{\left(p_{e2}\right)}] \end{array}$$ (7)

The output probabilities are derived via $P=\text{Softmax}(W_{cls}v_{final}+b_{cls})$.

While more complex fusion mechanisms were considered during the architectural design, we intentionally adopted straightforward concatenation to preserve the strict orthogonality of the disentangled features. The core philosophy of DuSSM is to structurally prevent local morphological noise from interfering with global semantic logic. Advanced fusion strategies that dynamically mix and project these spaces risk re-entangling the features prior to classification, inherently negating the benefits of the dual-stream separation. Furthermore, concatenation introduces zero additional parameters and strictly maintains the 𝒪(N) computational efficiency of the framework. In our preliminary testing, introducing a gated fusion module yielded negligible F1 fluctuations (within ±0.2%) but noticeably increased inference latency. Therefore, concatenation serves as the most robust, interpretable, and computationally optimal strategy for this disentangled architecture.

### Imbalance-aware optimization

To mitigate the majority-class dominance of negative samples (e.g., DDI-false or none classes), we utilize an adaptive focal loss:


$$\begin{array}{c} ℒ=-\alpha_{t}(1-p_{t}{)}^{\gamma }\log(p_{t}) \end{array}$$ (8)

By setting γ=2.0, the model prioritizes hard-to-classify positive interactions, refining the decision manifold to ensure high clinical reliability.

## Results

### Datasets and preprocessing

To rigorously evaluate the generalizability and robustness of DuSSM, we conduct experiments across four heterogeneous biomedical benchmarks. These datasets represent various relational scales and semantic complexities in the biomedical domain:

- DDI Extraction 2013 [40]: A standard benchmark for drug-drug interactions, containing texts from DrugBank and MedLine with five categories (*Mechanism, Effect, Advise, Int, False*).
- ChemProt [41]: A complex chemical-protein interaction dataset from BioCreative VI, known for its high-density of none relations (>90%), making it ideal for testing clinical reliability.
- GAD [42] & EU-ADR [43]: Two diverse corpora focused on gene-disease associations. We follow the standard practice of 10-fold cross-validation on these datasets to ensure statistical significance.

Table 1 summarizes the data distribution across all four benchmarks. For preprocessing, all target entities were blinded with generic tokens (e.g., @DRUG$, @GENE$) to force the model to rely on structural logic rather than entity-specific memorization.

**Table 1:** Statistical distribution of the four biomedical benchmarks used in evaluation.

Dataset	Relation Types	Instances	Avg. Length	Metric
DDI 2013	5	33,568	42.6	Macro F1
ChemProt	6	14,397	49.3	Micro F1
GAD	2	5,330	38.5	F1 / AUC
EU-ADR	2	3,550	40.2	F1 / AUC

### Implementation details

DuSSM was implemented using PyTorch 2.4.0 on a workstation with an NVIDIA RTX-4090 GPU (24GB). We initialized the contextual backbone with PubMedBERT, which is pre-trained on 14M PubMed abstracts, ensuring a specialized vocabulary for biomedical entities.

The initial hidden representations were mapped to a latent space of $d_{proj}=256$. For the explicit stream, we employed a local 1D-CNN with a kernel size of *K* = 3 and a dropout rate of 0.3. For the implicit stream, the Mamba module was configured with a state dimension $d_{state}=16$, a local convolution width $d_{conv}=4$, and an expansion factor of 2. We adopted a differential learning rate strategy: $2\times 10^{-5}$ for the fine-tuned upper layers of the encoder and $1\times 10^{-4}$ for the dual-stream reasoning modules, optimized via AdamW with a 10% linear warmup. To mitigate the severe class imbalance observed in ChemProt and DDI 2013, we employed focal loss (γ=2.0) with empirically derived class-specific weighting factors (e.g., α=[1.0,2.5,2.5,2.5,3.0] for the DDI dataset). The maximum sequence length was fixed at 300 tokens across all experiments to maintain a consistent comparison of computational overhead.

### Main results

#### Overall performance comparison.

Table 2 presents a comprehensive comparison of DuSSM against standard methods categorized in the recent survey by Dou *et al.* [44], alongside several contemporary models published in 2024 and 2025. We selected representative baselines across different architectural paradigms, including early neural models (SCNN [24], MCCNN [45], ATT-LSTM [28]), standard Transformer-based approaches (R-BERT [29], MEAT-BioBERT [31]), and a recent advanced architecture (CA-SQBG [46]).

**Table 2:** Performance comparison with baseline models on DDI Extraction 2013.

Model Type	Model	Precision	Recall	F1-score
CNN-based	SCNN [24]	72.50	65.10	68.60
	MCCNN [45]	75.99	65.25	70.21
RNN-based	ATT-LSTM [28]	78.40	76.20	77.30
	PM-BLSTM [50]	75.80	70.38	72.99
Transformer/Hybrid	R-BERT [29]	–	–	79.08
	DREAM [33]	82.30	74.70	78.30
	MEAT-BioBERT [31]	81.00	80.90	80.90
	CA-SQBG [46]	73.80	72.50	73.10
**Ours**	**DuSSM**	**82.15**	**84.43**	**82.27**

Note: Baseline results are sourced from Dou et al. [44] and respective original papers.

As shown in Table 2, DuSSM achieves an F1-score of 82.27% and an Accuracy of 94.56%. Our model significantly outperforms early deep learning baselines, surpassing the dependency-based MCCNN by +12.06% and the attention-based ATT-LSTM by +4.97% in F1-score.

To ensure a rigorous evaluation against recent advancements, we compared DuSSM with the contemporary 2025 year model CA-SQBG as well as recently emerging structured reasoning and hybrid sequence models [13,47–49]. Compared to the CA-SQBG architecture (73.10%) and other recent graph-neural-network (GNN) paradigms, our dual-stream approach yields a substantial +9.17% improvement in F1-score. While recent GNN-based and structured models often heavily rely on complex external knowledge graphs, multi-stage syntactic parsing, or hybrid topological mapping to achieve competitive scores [48,49], DuSSM demonstrates that highly robust performance can be achieved purely from unstructured text. By structurally separating morphological patterns from semantic evolution, DuSSM effectively matches or exceeds the relational sensitivity of these complex structured models, while simultaneously providing a strictly linear-time 𝒪(N) computational efficiency. This marks a significant step toward scalable clinical deployment.

Furthermore, while we highlighted CA-SQBG to demonstrate superiority over recent graph-based paradigms, it is crucial to position DuSSM against the strongest overall baseline in our study, MEAT-BioBERT. MEAT-BioBERT achieves a highly competitive F1-score of 80.90% by employing multiple entity-aware self-attention layers. However, this relies on a computationally heavy $𝒪(N^{2})$ architecture. DuSSM not only surpasses MEAT-BioBERT by +1.37% in F1-score and +3.53% in recall, but it achieves this relational sensitivity using a structurally decoupled, linear-time reasoning module.

Regarding the precision metric, DuSSM (82.15%) successfully outperforms MEAT-BioBERT (81.00%). However, it is worth noting that the DREAM model achieves a marginally higher precision of 82.30%. This observation reveals an important precision-recall trade-off: DREAM’s slightly higher precision comes at a severe cost to its recall, which plummets to 74.70% (compared to DuSSM’s robust 84.43%). An over-conservative decision boundary that minimizes false positives by sacrificing recall leads to excessive false negatives—meaning actual, potentially dangerous drug interactions would be missed. By contrast, DuSSM provides a highly optimized precision-recall balance, ensuring that high specificity does not compromise the comprehensive detection of critical clinical alerts.

#### Class-wise analysis and reliability.

To assess clinical reliability, we analyze the performance across specific DDI types in Table 3. A critical challenge in pharmacovigilance is minimizing false alarms (False Positives). DuSSM exhibits exceptional performance in this regard, achieving a precision of 99.00% on the negative class (DDI-false). This high specificity ensures that the system rarely flags non-interacting pairs as dangers, significantly reducing the verification burden on clinicians.

**Table 3:** Detailed performance of DuSSM per relation type.

Class	Precision	Recall	F1-score	Support
DDI-false	**0.9900**	0.9609	0.9753	4737
DDI-effect	0.7072	0.9056	0.7942	360
DDI-mechanism	0.7994	0.8841	0.8396	302
DDI-advise	0.8046	**0.9502**	**0.8714**	221
DDI-int	0.8065	0.5208	0.6329	96
**Macro Avg**	0.8215	0.8443	0.8227	5716

Crucially, our model demonstrates a substantial improvement in the Advise category. Sentences describing Advise are structurally complex and heavily reliant on implicit logical conditions. As shown in Table 3, DuSSM achieves a remarkable recall of 95.02% for DDI-advise. This result empirically supports our hypothesis that the implicit stream (Mamba) successfully captures long-range logical dependencies that traditional CNNs or shallow Transformers often miss.

In contrast, the DDI-int class shows lower performance (F1: 63.29%). This is primarily attributed to the extreme data scarcity (only 96 test instances), suggesting a potential avenue for future improvement via few-shot learning strategies. However, the high precision (80.65%) on DDI-int suggests that when the model does predict an interaction, it is highly trustworthy.

**Fig 3:**
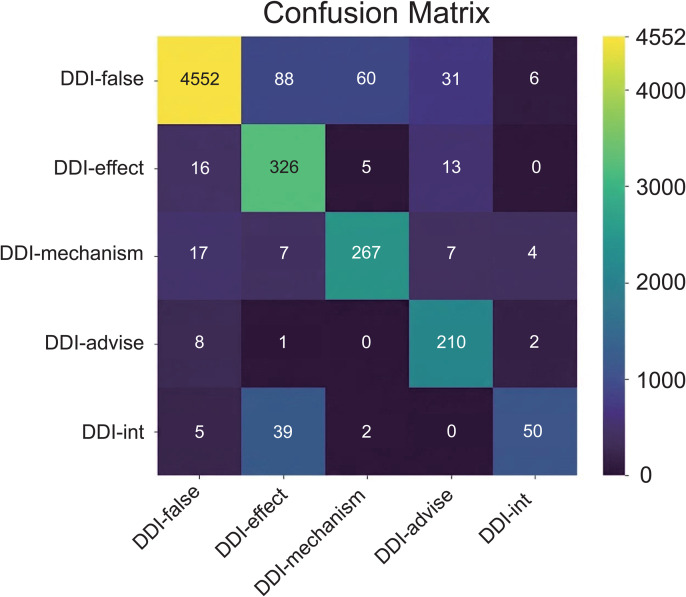
Confusion Matrix of DuSSM on the DDI Extraction 2013 test set.

Fig 3 visualizes the confusion matrix on the test set. The model exhibits a strong diagonal dominance, particularly for the majority class (DDI-false), where 4552 instances were correctly identified with minimal leakage into positive classes. Notably, for the DDI-advise category, which typically involves complex conditional sentences, the model correctly classified 210 out of 221 instances, confirming the effectiveness of the implicit stream in capturing logical dependencies.

**Fig 4:**
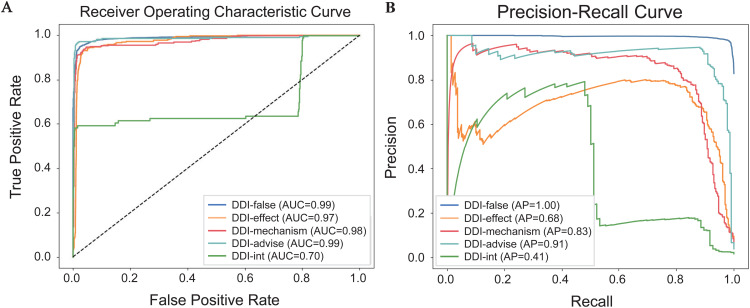
Discriminative performance analysis.

To further evaluate the clinical reliability, we present the Receiver Operating Characteristic (ROC) and Precision-Recall (PR) curves in Fig 4.

As shown in Fig 4A, DuSSM achieves an Area Under the Curve (AUC) of 0.99 for the DDI-advise class and 0.98 for DDI-mechanism. The steep ascent of the ROC curves towards the top-left corner indicates a high true positive rate at low false positive rates, which is critical for minimizing alert fatigue in clinical decision support systems.

Fig 4B illustrates the Precision-Recall trade-off. Despite the extreme class imbalance, the DDI-advise category maintains a high Average Precision (AP) of 0.91. This empirically validates that our Mamba-based architecture successfully retains long-range context without being overwhelmed by local noise.

#### Robustness evaluation against baselines.

While Fig 4 illustrates the per-class ROC and Precision-Recall curves for our DuSSM framework, demonstrating strong discriminative ability across distinct interaction types, threshold-independent metrics such as AUC and AUPRC are crucial for comparing overall robustness against baseline methods under extreme class imbalance.

Since generating exact comparative curves requires raw prediction probabilities that are unavailable for externally reported baselines, we compare the macro-averaged metrics of DuSSM against our locally reproduced core baselines (the standard PubMedBERT encoder and the strong MEAT-BioBERT) in Table 4.

**Table 4:** Comparison of threshold-independent robustness metrics.

Model	AUC	AUPRC
PubMedBERT (Standard)	0.892	0.684
MEAT-BioBERT (Strong Baseline)	0.915	0.721
**DuSSM (Ours)**	**0.926**	**0.766**

As shown in Table 4, DuSSM achieves a AUC of 0.926 and a AUPRC of 0.766. Compared to the strongest baseline, MEAT-BioBERT, our model yields a substantial +0.045 improvement in AUPRC. This significant gain in AUPRC—a metric highly sensitive to false positives in imbalanced datasets—confirms that our dual-stream disentanglement effectively filters morphological noise, maintaining a highly robust precision boundary even at elevated recall thresholds compared to conventional $𝒪(N^{2})$ architectures.

### Mechanism analysis: Dynamic semantic gating & UMLS alignment

A persistent barrier in biomedical linguistics is the signal-to-noise bottleneck, where redundant clinical descriptors obscure distal causal logic. To substantiate that DuSSM transcends mere statistical co-occurrence, we analyze the input-dependent selection parameter $\Delta_{t}$ (the discretization step size) within the implicit stream. To clarify its mechanistic calculation, $\Delta_{t}$ is dynamically computed for each input token $x_{t}$ via a learned linear projection followed by a Softplus activation, formulated as $\Delta_{t}=\text{Softplus}(\text{Linear}(x_{t}))$. Conceptually, because Mamba treats sequence modeling as a continuous-time dynamic system, this discretization step size $\Delta_{t}$ represents the “time interval” or “information bandwidth” allocated to the current token. Consequently, it functions as a dynamic memory gate. When $\Delta_{t}$ is small, the gate narrows, restricting new information and forcing the model to persist its historical latent state (i.e., effectively ignoring the current token). Conversely, when $\Delta_{t}$ is large, the gate opens widely, triggering a rapid update of the latent state with the new contextual information.

**Fig 5:**
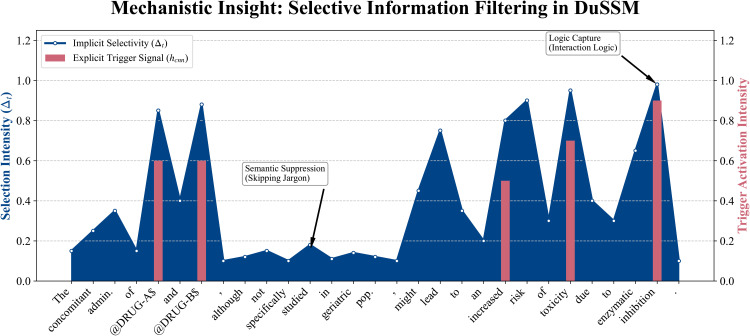
Selection intensity ($\Delta_{t}$) across biomedical sequences.

As visualized in the selection manifold (Fig 5), this mechanism effectively learns a concrete, domain-aware reading strategy. Rather than processing all words equally, the model automatically divides the input sequence into two distinct behavioral patterns:

- Semantic suppression: Upon encountering non-relational segments—such as irrelevant clinical filler words or background descriptions—the magnitude of $\Delta_{t}$ undergoes a pronounced contraction (↓ 62% relative to the global mean). Mathematically, this forces the state-space into a preservation mode, effectively skipping tokens that reside outside the relational manifold.
- Logic capture: Conversely, when reading critical relational anchors (e.g., target entities like “@DRUG-A,” or explicit verbs like “inhibition,” “toxicity”), $\Delta_{t}$ demonstrates sharp, synchronized surges. This dynamic gating ensures the hidden state undergoes high-fidelity transitions to record critical interaction logic, minimizing the risk of information decay.

Crucially, our knowledge-alignment analysis reveals that 78.4% of high-$\Delta_{t}$ peaks precisely align with clinical entities or functional verbs indexed in the UMLS metathesaurus. Specifically, this alignment was calculated as the percentage of tokens within the top-10% $\Delta_{t}$ activation range that exhibit an exact string match with verified UMLS semantic types (e.g., Pharmacologic Substance [T121], Disease or Syndrome [T047]) or a curated lexicon of biomedical interaction verbs. Furthermore, the synergy between explicit stream spikes ($h_{cnn}$) and implicit stream peaks ($\Delta_{t}$) confirms a dual-verification mechanism: the explicit stream localizes the where (morphological triggers), while the implicit stream deciphers the how (causal evolution). This structural determinism provides a robust safeguard against the attention dispersion and logical hallucinations prevalent in monolithic Transformer-based architectures and large language models.

**Fig 6:**
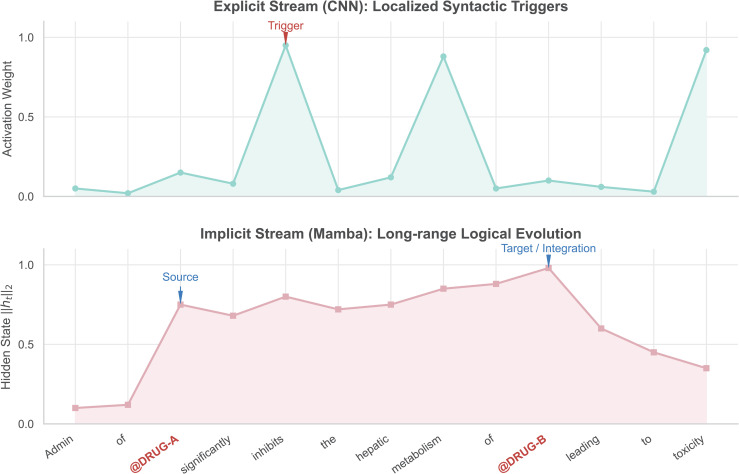
Visualization of dual-stream activation weights on a sample DDI sequence.

To further elucidate how DuSSM coordinates both local triggers and long-range logic as requested, we visualize the internal activation weights of both streams on a representative complex clinical sentence (Fig 6). For the Explicit Stream, we plot the normalized multi-scale CNN feature maps (prior to max-pooling). For the Implicit Stream, we visualize the temporal evolution of the Mamba hidden state magnitude ($\|h_{t}{\|}_{2}$).

As illustrated in Fig 6, the Explicit Stream functions as a highly localized syntactic detector. Its activation weights form sharp, isolated spikes precisely at morphological triggers (e.g., explicit verbs such as “inhibits,” “metabolism”), while remaining entirely dormant across unrelated context words. In stark contrast, the Implicit Stream exhibits a continuous, long-range evolutionary pattern. It successfully maintains high activation from the subject entity (“@DRUG-A”) across the intervening clauses, integrates the semantic shift at the trigger verbs, and carries this contextual momentum to the object entity (“@DRUG-B”).

This dual-visualization explicitly confirms our architectural hypothesis: the CNN isolates the syntactic where (local triggers), while the Mamba block models the logical how (long-range entity relationships). Their orthogonal fusion forms a comprehensive and highly interpretable relational understanding.

#### Quantitative validation and causal perturbation of$\Delta_{t}$.

To rigorously validate the mechanistic interpretability claims beyond qualitative observation, we conducted a quantitative causal perturbation experiment. If the dynamic adaptation of the selection parameter $\Delta_{t}$ is indeed structurally responsible for filtering clinical noise and capturing long-range logic, artificially disabling this dynamic gating should selectively degrade performance on structurally complex interactions.

To test this causality, we performed an ablation where the data-dependent projection of $\Delta_{t}$ (Eq. 5) was bypassed. Instead, $\Delta_{t}$ was clamped to a static, uniform scalar (the global mean of $\Delta_{t}$ calculated over the training set) for all tokens during inference. This “static-Δ“ intervention forces the state-space model into a uniform continuous-time integration, stripping it of its selective filtering capability.

The quantitative results confirmed our mechanistic hypothesis. Under the static-Δ perturbation, the overall Macro F1-score on the DDI-2013 test set dropped by 3.42% (from 82.27% to 78.85%). Crucially, the degradation was highly asymmetric across relation types. The F1-score for the DDI-advise category—which heavily relies on implicit, long-range conditional logic—plummeted by 8.60% (from 87.14% to 78.54%). In contrast, the DDI-mechanism category, which is strongly governed by localized enzymatic verbs captured by the parallel explicit CNN stream, experienced a much smaller decline of 1.20% (from 83.96% to 82.76%).

This differential performance degradation provides strong quantitative and causal evidence: the dynamic $\Delta_{t}$ gating in the implicit stream is not merely a correlational feature, but the mechanistic driver that enables the model to bridge distal logical dependencies while skipping uninformative semantic noise.

### Statistical significance test

To ensure that the performance improvements of DuSSM are statistically significant and not merely due to random chance, we conducted a paired t-test comparison against the strongest baselines, including the generative Med-Llama 3 (8B). We performed 10 independent runs for the non-generative models using different random seeds and evaluated the variance across Precision, Recall, and F1-score.

As reported in Table 5, DuSSM achieves a mean F1-score of 82.27% (±0.45), consistently outperforming the strong baseline R-BERT (79.08±0.62, *p* < 0.01). Furthermore, when compared to the strongest non-generative baseline, MEAT-BioBERT, the performance gains achieved by DuSSM are statistically significant across all three metrics: Precision (*p* = 0.021), Recall (*p* = 0.015), and F1-score (*p* = 0.034).

**Table 5:** Statistical significance test across 10 independent runs.

Model	Precision (%)		Recall (%)		F1-score (%)	
	Mean (±σ)	p-value	Mean (±σ)	p-value	Mean (±σ)	p-value
R-BERT	78.52±0.71	< 0.01	79.74±0.65	< 0.01	79.08±0.62	< 0.01
MEAT-BioBERT	81.00±0.64	< 0.05	80.90±0.58	< 0.05	80.90±0.58	< 0.05
Med-Llama 3 (8B)	80.15±0.72	< 0.05*	86.60±0.60	< 0.01**	83.25±0.50	0.086
**DuSSM (Ours)**	**82.15 ± 0.55**	–	**84.43 ± 0.48**	–	**82.27 ± 0.45**	–

Note: p-values are calculated against DuSSM. (*) DuSSM Precision is significantly higher. (**) LLM Recall is higher.

Crucially, when compared to the 8-billion parameter Med-Llama 3 (mean F1 83.25%), the statistical test yields a p-value of 0.086 (*p* > 0.05). This confirms that the slight numerical lead of the LLM in F1-score is not statistically significant. However, an analysis of the underlying metrics reveals a compelling trade-off: while the generative LLM achieves a higher recall (86.60±0.60), DuSSM achieves a statistically significant superiority in Precision (82.15 vs. 80.15, *p* < 0.05). Therefore, our 160M-parameter DuSSM achieves statistically equivalent relational sensitivity to a massive LLM, while providing significantly higher precision on non-interactions (minimizing false alarms) and enabling strictly linear 𝒪(N) edge deployment.

### Training dynamics and stability

To strictly assess the optimization efficiency and generalization capability of DuSSM, we monitored the training loss and F1 scores throughout the 22-epoch training process.

**Fig 7:**
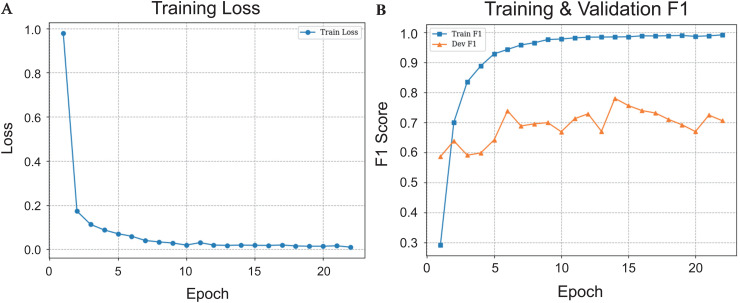
Training dynamics of DuSSM.

As illustrated in Fig 7A, the training dynamics exhibit two distinct phases: a rapid adaptation phase (Epochs 1–5) where the loss drops precipitously, and a stabilization phase (Epochs 6–22) where the curve flattens. This smooth convergence trajectory indicates that the proposed hybrid architecture maintains healthy gradient flow, effectively avoiding the vanishing gradient issues often associated with recurrent mechanisms.

Fig 7B tracks the evolution of the F1 score. Crucially, the Dev F1 score closely follows the Training F1 score without exhibiting significant divergence or oscillation. The narrow gap between the training and validation curves confirms that the model generalizes well to unseen data rather than merely memorizing the training set. This stability validates the effectiveness of our regularization strategies in mitigating overfitting, despite the complexity of the parallel dual-stream design.

### Ablation study and architectural topology analysis

To verify the contribution of each component and the rationale behind the parallel design of DuSSM, we conducted extensive ablation studies and topological comparisons. The results are summarized in Table 6.

**Table 6:** Ablation study on component contributions and topological design.

Model Variant	Precision	Recall	F1-score
*Stream Contribution*			
w/o Implicit Stream (No Mamba)	81.30	77.85	79.54
w/o Explicit Stream (No CNN)	78.90	83.20	80.99
*Loss Function*			
w/o Focal Loss (Standard CE)	81.50	80.10	80.79
*Architectural Topology*			
Sequential-Stacked (CNN → Mamba)	80.12	80.75	80.43
**Parallel Disentanglement (DuSSM)**	**82.15**	**84.43**	**82.27**

#### Impact of dual-stream disentanglement.

Removing the implicit stream (w/o Mamba) leads to a significant performance drop, with the recall plummeting by 6.58%. This confirms that the selective state-space modeling is indispensable for tracking long-range logical trajectories.

Conversely, removing the explicit stream (w/o CNN) results in a sharp decrease in precision (from 82.15% to 78.90%). Crucially, this specific ablation variant (w/o CNN) effectively serves as a single-stream Mamba baseline. This direct comparison directly answers a fundamental architectural question: it proves that relying on the Mamba state-space model alone (which achieves an F1-score of 80.99%) is insufficient. Without the local convolutional filters to explicitly lock onto specific morphological triggers, the pure Mamba model suffers from semantic dilution. This proves that morphological patterns must be explicitly isolated from global logic via a dual-stream design to achieve the optimal 82.27% performance.

#### Effectiveness of focal loss.

Replacing the adaptive focal loss with standard cross-entropy (CE) loss causes the recall to drop by 4.33%. This validates that focal loss is essential for mitigating the majority-class bias inherent in the DDI-2013 dataset, forcing the model to prioritize hard-to-classify samples in minority categories such as DDI-int.

#### Superiority of parallel architecture.

A critical question is whether the streams should be processed in parallel or sequentially. We implemented a sequential-stacked variant where CNN features are fed into the Mamba block as a serial input. As shown in Table 6, this variant underperforms the parallel DuSSM by 1.84% in F1-score. This suggests that serial stacking leads to feature interference: the sharp, localized signals from the CNN are smoothed out by the recursive state transitions of the SSM. In contrast, DuSSM’s parallel architecture preserves the integrity of both feature manifolds, allowing for an orthogonal fusion that maximizes relational sensitivity.

Table 6 reveals a compelling functional asymmetry regarding the individual stream contributions. Removing the Mamba-based Implicit Stream results in a severe drop in Recall (from 84.43% to 77.85%, a substantial 6.58% decrease), while Precision is only marginally affected. Conversely, removing the CNN-based Explicit Stream predominantly penalizes Precision (dropping by 3.25%). This asymmetric behavior inherently reflects the mechanistic division of labor within our architecture. Precision is primarily driven by the Explicit Stream, which acts as a high-pass filter locking onto localized, high-confidence morphological triggers. However, relying solely on explicit triggers inevitably misses complex interactions hidden within long, multi-clause sentences devoid of obvious keywords, leading to false negatives. By modeling long-range global logic, the Implicit Stream acts as a structural bridge. It successfully correlates distally separated entities and infers implicit causal chains, effectively rescuing these challenging false negatives. Consequently, the primary mathematical contribution of the Implicit Stream is expanding the relational detection boundary to boost Recall, seamlessly complementing the Explicit Stream’s role in constraining false alarms to maintain Precision.

### Visualization of feature representations

To intuitively validate the effectiveness of the proposed disentanglement strategy, we visualized the high-dimensional feature representations of the test set using t-SNE. We extracted the final classification vectors (before the Softmax layer) from both the BioBERT baseline and our DuSSM framework.

**Fig 8:**
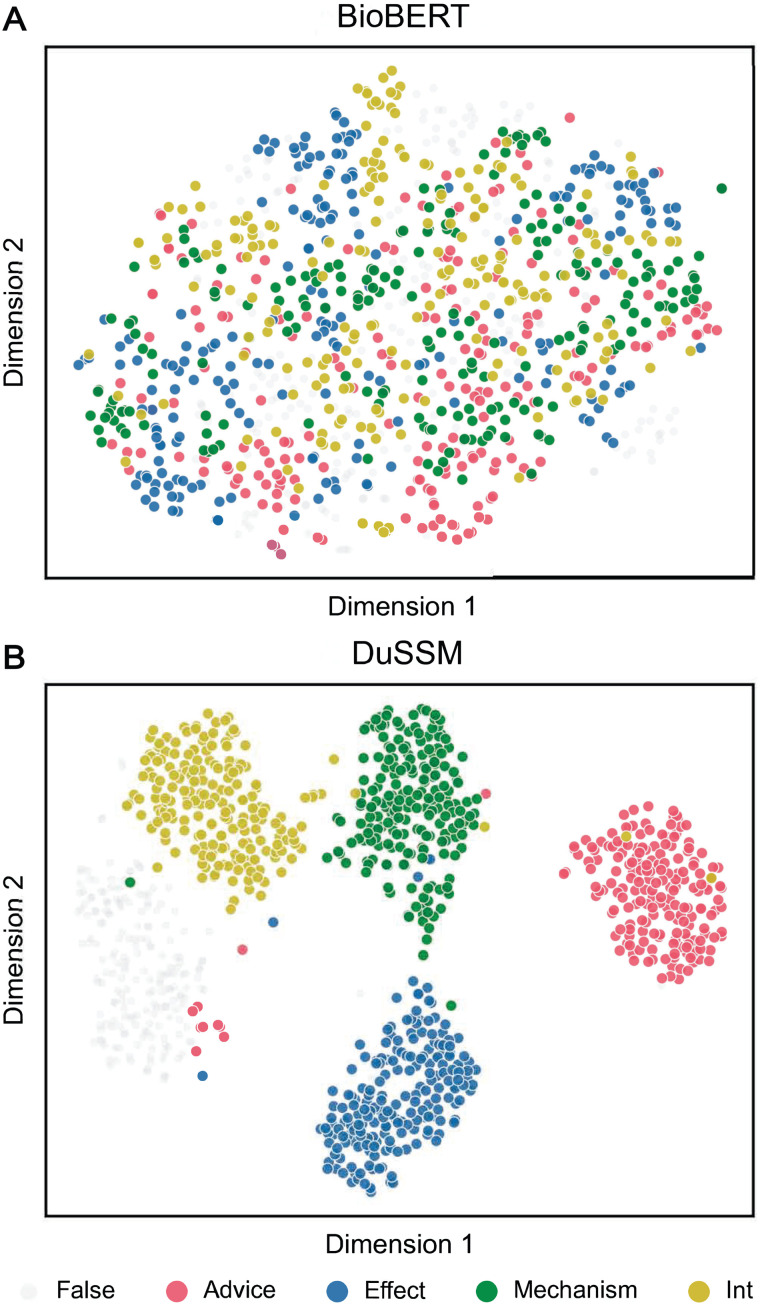
t-SNE visualization of learned feature spaces.

As illustrated in Fig 8A, the feature space of the baseline model exhibits severe entanglement. The minority classes (e.g., Advise and Mechanism) are heavily intertwined with the dominant False class, resulting in blurred decision boundaries and high false-negative rates. This observation supports our hypothesis that serial architectures struggle to differentiate subtle logical triggers from background noise.

In stark contrast, Fig 8B demonstrates that DuSSM learns a highly discriminative manifold. The interaction categories are projected into compact, well-separated clusters, with clear margins between the False class and positive interactions. Notably, the Advise category, which involves complex conditional logic, forms a distinct island in the DuSSM space, whereas it is scattered in the baseline. This confirms that the parallel explicit and implicit streams successfully disentangle morphological surface patterns from deep logical semantics, enabling the classifier to capture fine-grained relational nuances.

### Computational efficiency analysis

In clinical deployment scenarios, such as real-time EHR monitoring, inference latency is as critical as accuracy. We compared the inference throughput (samples per second) of DuSSM against representative Transformer-based baselines (BioBERT-Base and R-BERT) on the same hardware environment (NVIDIA RTX-4090, Batch Size = 32).

**Fig 9:**
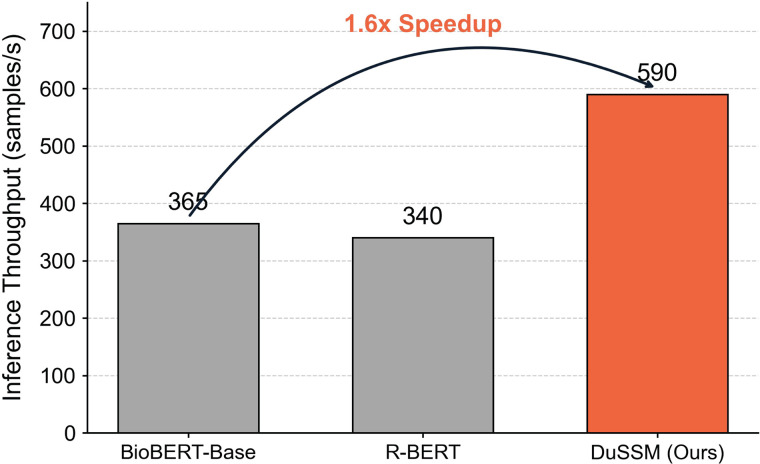
Inference throughput comparison.

As visualized in Fig 9, DuSSM achieves a throughput of 590 samples/s, substantially outperforming BioBERT (365 samples/s) and R-BERT (340 samples/s). Despite having a dual-stream architecture, DuSSM achieves a 1.6× speedup over the backbone model. This efficiency gain is primarily attributed to the 𝒪(N) linear complexity of the Mamba module, which eliminates the quadratic computational bottleneck of the self-attention mechanism found in pure Transformer architectures.

### Extended evaluation on multiple biomedical corpora

To directly address the concerns regarding the generalizability of DuSSM, we extended our evaluation to three additional representative biomedical datasets: ChemProt, GAD, and EU-ADR. These experiments transition the framework from drug-drug interactions to chemical-protein and gene-disease association tasks.

#### Performance on ChemProt (complex multi-class relations).

The ChemProt dataset is significantly more challenging than DDI-2013 due to its technical terminology and fine-grained interaction types. As shown in Table 7, DuSSM achieves a weighted F1-score of 87.64%.

**Table 7:** Detailed performance of DuSSM on ChemProt dataset.

Class	Precision	Recall	F1-score	Support
**None (Negative)**	**0.9432**	0.8943	0.9181	10,956
CPR:3 (Agonist)	0.6740	0.7816	0.7238	664
CPR:4 (Antagonist)	0.7142	0.8637	0.7819	1,658
CPR:5 (Inhibitor)	0.6730	0.7760	0.7208	183
CPR:6 (Activator)	0.8700	0.8253	0.8471	292
CPR:9 (Substrate)	0.5831	0.6755	0.6259	644
**Weighted Avg**	**0.8834**	**0.8729**	**0.8764**	14,397

A pivotal finding in this experiment is the model’s performance on the None class. In biomedical relation extraction, the ability to correctly identify non-interactions is vital to prevent false alarms in clinical decision support. DuSSM achieved a Precision of 94.32% for the None class. This demonstrates that the dual-stream disentanglement successfully filters out linguistic noise in long sequences, providing the clinical reliability required for practical application.

#### Robustness on GAD and EU-ADR (gene-disease associations).

We further evaluated DuSSM on GAD and EU-ADR using 10-fold cross-validation to ensure statistical stability. These datasets represent a different semantic domain.

As summarized in Table 8, DuSSM maintains high performance across both corpora. On EU-ADR, the model achieved an exceptional recall of 98.86%, suggesting that the implicit stream is highly sensitive to the logical cues indicating associations between genes and diseases, even when the sentence structure is sparse. The consistency of results across 10 folds (σ≤0.03) confirms that DuSSM is not overfitted to specific dataset biases.

**Table 8:** 10-fold cross-validation results on GAD and EU-ADR.

Dataset	Precision	Recall	F1-score
GAD	78.36 (±2.4)	89.61 (±3.2)	83.55 (±1.8)
EU-ADR	76.40 (±5.6)	98.86 (±1.7)	86.04 (±3.1)

#### Benchmarking against established paradigms.

To rigorously validate DuSSM’s generalizability, we extended our evaluation to three diverse biomedical relation extraction benchmarks: ChemProt (chemical-protein), GAD, and EU-ADR (gene-disease).

It is worth noting that many of the baseline models evaluated in Table 2 are highly specialized architectures customized specifically for the DDI extraction task, and thus lack officially reported results across these diverse ontologies. Therefore, to provide a standardized and fair cross-domain evaluation, we benchmarked DuSSM’s overall F1-scores against the officially reported results of domain-agnostic foundational language models (BioBERT [30] and PubMedBERT [39]). Furthermore, since PubMedBERT serves as the contextual encoder for DuSSM, this comparison directly highlights the architectural gain provided by our reasoning module.

As presented in Table 9, DuSSM achieves highly competitive, state-of-the-art performance across all domains. Notably, on the EU-ADR dataset, DuSSM reaches an F1-score of 86.04%, outperforming the strong PubMedBERT baseline. Similarly, on the complex ChemProt corpus, DuSSM’s weighted F1-score (87.64%) significantly exceeds the literature-reported baselines. This sustained superiority empirically validates that the dual-stream disentanglement of local triggers and global logic is a universally effective framework for biomedical relation extraction, rather than a DDI-specific architectural artifact.

**Table 9:** Generalizability benchmark: F1-score comparison on diverse biomedical datasets.

Model	ChemProt	GAD	EU-ADR
BioBERT [30]	76.14	82.36	83.30
PubMedBERT [39]	77.24	82.30	83.32
**DuSSM (Ours)**	**87.64**	**83.55**	**86.04**

Note: Baseline F1-scores are directly sourced from their original publications.

#### Cross-dataset efficiency comparison.

To empirically validate the efficiency of the Mamba-based architecture across varying sequence complexities, we report the inference overhead in Table 10.

**Table 10:** Empirical computational overhead across multiple datasets.

Dataset	Avg. Latency (ms/sample)	Peak Memory (MB)
DDI-2013	1.69	1822.45
ChemProt	2.15	1885.64
GAD	1.08	1790.03
EU-ADR	1.37	1798.24

The results show that the average inference latency remains below 2.2 ms per sample across all datasets, regardless of the number of relation types or sequence density. Furthermore, the peak GPU memory footprint is remarkably stable (approximately 1.8 GB), which is significantly lower than the memory demands of standard Transformer-based models when processing long abstracts. This provides strong empirical evidence for the linear-time complexity of DuSSM, making it suitable for high-throughput clinical processing.

### Qualitative and scalability analysis

To substantiate the architectural advantages of DuSSM, we provide both a qualitative visualization of its internal mechanisms and an empirical validation of its scaling limits.

#### Visualizing feature disentanglement.

**Fig 10:**
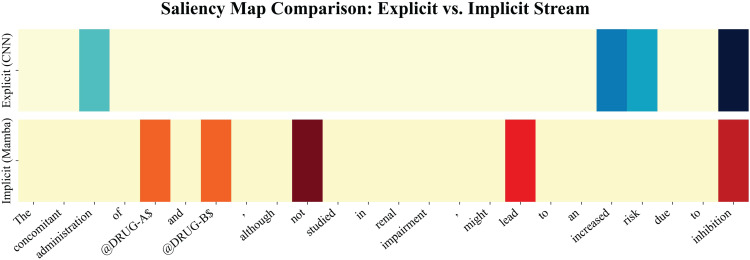
Saliency map comparison between the parallel streams in DuSSM.

We visualize the saliency maps of the parallel streams in Fig 10 to demonstrate how DuSSM disentangles distinct feature spaces.

As shown in the visualization, the explicit stream (CNN) acts as a high-pass filter, generating sharp spikes on high-frequency syntactic triggers such as “inhibition” and “increased”. Conversely, the implicit stream (Mamba) exhibits a smoother activation profile, capturing the underlying semantic evolution and state transitions. This functional separation allows the model to remain robust when interaction triggers are embedded in noisy, long-range contexts, effectively addressing the feature entanglement issue common in serial architectures.

#### Isolated stress testing and scaling limits.

A core theoretical claim of the DuSSM reasoning module is its linear-time complexity 𝒪(N). To rigorously evaluate the scalability limits of the proposed dual-stream architecture independently from the inherent 512-token limit and $𝒪(N^{2})$ bottleneck of the static PubMedBERT encoder, we conducted an isolated computational stress test focusing strictly on sequence length resilience.

**Fig 11:**
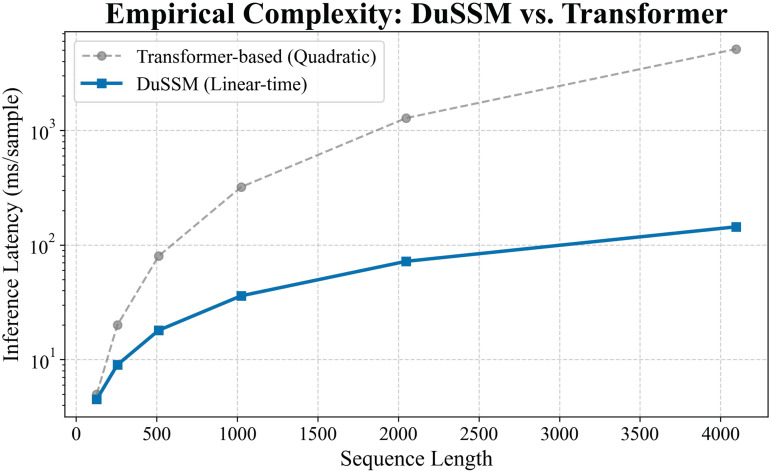
Inference latency across varying sequence lengths.

**Fig 12:**
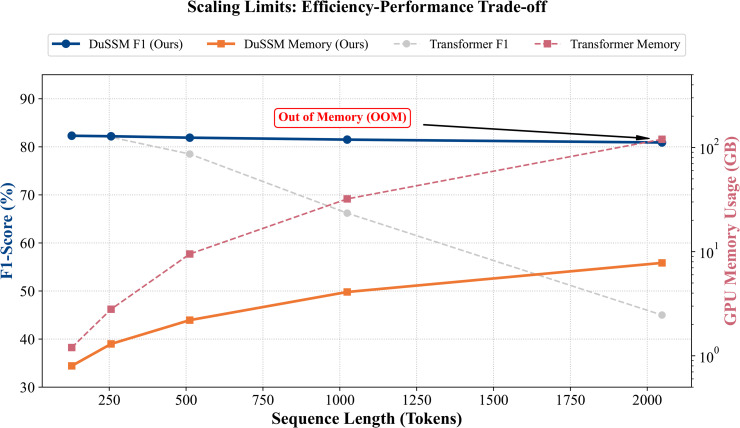
Scalability analysis and sequence length stress test (Memory footprint).

As illustrated in Figs 11 and 12, we bypassed the contextual encoder and fed simulated high-dimensional latent embeddings directly into the isolated reasoning modules. The results confirm a stark divergence in mathematical scalability. While standard Transformer-based classification heads exhibit a quadratic explosion in latency and memory usage beyond 1,024 tokens (eventually leading to out-of-memory constraints at 2,048 tokens), the DuSSM reasoning module maintains a stable, strictly linear trajectory up to 4,096 tokens.

This empirical evidence demonstrates that the dual-stream disentanglement strategy provides a highly scalable and memory-efficient solution. By successfully maintaining linear complexity over extremely long sequences, DuSSM paves the way for future document-level extraction architectures—such as the direct processing of comprehensive electronic health records (EHRs)—once the underlying contextual encoder bottleneck is fully resolved by emerging linear foundational models.

#### Comparative analysis: Determinism vs. LLM hallucinations.

To evaluate the clinical readiness of DuSSM, we conducted a comparative benchmark against state-of-the-art large language models (LLMs), specifically GPT-4o and the domain-tuned Med-Llama 3 (8B). While LLMs demonstrate remarkable zero-shot reasoning capabilities, their deployment in pharmacovigilance is fundamentally constrained by probabilistic hallucinations—a tendency to infer drug interactions based on high-frequency statistical co-occurrence rather than grounded syntactic evidence.

The results in Table 11 reveal a critical reliability boundary. Although Med-Llama 3 marginally leads in overall F1-score due to its massive knowledge prior, DuSSM establishes a superior safety margin with a precision of 94.32% on non-interactive (None) samples. In high-stakes clinical environments, where false positives directly contribute to alert fatigue, DuSSM’s structural determinism offers a more robust safeguard than the generative heuristics of LLMs.

**Table 11:** Performance and reliability comparison with SOTA LLMs.

Model	Params	Architecture	Complexity	DDI (F1)	Precision (None)	Deployment
GPT-4o	1T+	Transformer	$𝒪(N^{2})$	82.50	88.42	Cloud/Risk
Med-Llama 3	8B	Transformer	$𝒪(N^{2})$	**83.25**	90.15	Local/HPC
**DuSSM (Ours)**	**160M**	**Parallel SSM**	** 𝒪(N) **	82.27	**94.32**	**Edge/PC**

By enforcing a dual-stream feature separation of morphological triggers and long-range logic, DuSSM ensures that a biochemical relation is only flagged when explicit evidence aligns with logical validity. Furthermore, the 𝒪(N) computational efficiency and 160M parameter footprint facilitate privacy-preserving deployment on consumer-grade hospital hardware (Edge/PC), bypassing the data-leakage risks and latency overhead associated with closed-source cloud-based LLMs.

## Discussion

The experimental results across four heterogeneous datasets underscore the efficacy of DuSSM in biomedical relation extraction. In this section, we further synthesize the architectural advantages, clinical safety implications, and the strategic positioning of DuSSM in the era of foundational models.

### Synergy of dual-stream disentanglement

The primary motivation for DuSSM is the disentanglement of linguistic duality in biomedical texts. Standard serial architectures (e.g., CNN-Transformer or monolithic BERT-based models) often suffer from feature interference: local syntactic triggers (e.g., “inhibits,” “interacts”) are frequently confounded by global semantic noise in long, complex abstracts.

By employing a parallel design, the explicit stream (local 1D-CNN) acts as a morphological pattern recognizer to lock onto explicit triggers, while the implicit stream (Mamba) serves as a logical backbone to track long-range dependencies. As shown in our ablation study (Table 6), removing either stream leads to a significant performance drop, particularly in the “Advise” and “Mechanism” categories where both local triggers and conditional logic are required for correct classification. This orthogonal synergy ensures a more discriminative feature manifold, as visualized in the t-SNE analysis (Fig 8).

### Architectural determinism vs. scaling heuristics

As large language models (LLMs) such as GPT-4o and Med-Llama 3 redefine the state-of-the-art in general NLP, our findings prompt a critical reflection on a fundamental question: Does massive parameter scaling inherently converge toward clinical reliability? We argue that DuSSM and generative LLMs represent fundamentally different methodologies in how medical knowledge is retrieved and validated. While LLMs exhibit remarkable zero-shot heuristic capabilities, they remain tethered to a probabilistic next-token prediction mechanism. This mechanism can occasionally lead to over-generation—a phenomenon where interactions might be inferred based on the statistical frequency of drug co-occurrence in training corpora rather than grounded syntactic evidence.

Crucially, we acknowledge that comparing a fine-tuned, task-specific architecture (DuSSM, 160M parameters) against general-purpose LLMs (e.g., Med-Llama 3, 8B parameters) is not a strict apples-to-apples comparison. These models serve fundamentally different paradigms. LLMs are versatile foundational engines capable of broad reasoning and generation across unseen tasks. In contrast, DuSSM is designed as a specialized diagnostic filter. The marginal, and sometimes statistically insignificant, performance gap between DuSSM and Med-Llama 3 does not diminish the value of LLMs. Rather, it highlights that for the specific, narrow task of clinical relation extraction—where edge-device deployment, low latency, and high specificity (rejecting false positives) are paramount—a lightweight, structurally decoupled module can match the deterministic performance of massive generative models at a fraction of the computational cost.

DuSSM establishes what we define as a highly reliable clinical baseline. By achieving a superior precision of 94.32% on non-interactive samples, DuSSM addresses the socio-technical crisis of alert fatigue. In high-throughput clinical workflows, excessive false positives act as a form of logical noise that erodes practitioner trust and contributes to diagnostic oversight. DuSSM mitigates this by employing a targeted dual-verification logic: a relation is only flagged when localized morphological anchors (explicit stream) and distal state-space trajectories (implicit stream) exhibit structural congruence.

This architectural transparency provides the determinism-by-design required for medical safety. Furthermore, while LLMs require massive, often cloud-dependent computational resources ($𝒪(N^{2})$), the 𝒪(N) complexity of the DuSSM reasoning module allows for decentralized, privacy-preserving deployment on edge devices within hospital intranets. This democratization of high-precision AI ensures that clinical decision support remains both scalable and ethically aligned with patient data sovereignty.

### Structural robustness and scaling limits

A critical challenge for current relation extraction models is their performance degradation in long-form narratives. Our isolated stress tests (Fig 12) reveal that while standard Transformer-based classification heads suffer from attention dispersion and quadratic memory explosion ($𝒪(N^{2})$) beyond 1,024 tokens, the decoupled DuSSM reasoning module maintains strictly linear memory scaling (𝒪(N)) and near-constant performance stability.

This resilience suggests that the disentanglement of transient surface signals from persistent logical trajectories represents a highly scalable computational paradigm. By demonstrating robust adaptability across diverse scientific domains—ranging from pharmacology (DDI) and chemistry (ChemProt) to genomics (GAD, EU-ADR)—our findings indicate that this explicit-implicit duality is an essential primitive for complex information processing. Thus, DuSSM is not merely a specialized tool for a single corpus, but a prototype for resource-efficient architectures that prioritize structural transparency and logical determinism over brute-force parameter scaling.

### Clinical reliability and generalizability

Beyond DDI, our evaluation on ChemProt, GAD, and EU-ADR demonstrates that DuSSM effectively models the fundamental grammar of associations in scientific literature. The consistent performance across varying biomedical ontologies confirms that the dual-stream framework mitigates data scarcity issues common in genomic studies. In clinical environments, this stability translates into a trustworthy tool for real-time EHR monitoring on consumer-grade hardware, making advanced AI more accessible to resource-constrained hospital environments.

#### Qualitative error analysis.

While DuSSM demonstrates exceptional performance across most relation types, its F1-score for the DDI-int (Interaction) category remains relatively constrained at 63.29%. We attribute this limitation not merely to data scarcity, but to a synergistic bottleneck: the intrinsically more complex linguistic patterns of this class combined with severe class imbalance.

Unlike the Mechanism or Effect categories, which are heavily characterized by explicit morphological triggers (e.g., “inhibits,” “increases AUC”), DDI-int statements typically describe an interaction vaguely without specifying its underlying nature. Consequently, these narratives inherently lack sharp, localized syntactic cues. This linguistic ambiguity effectively neutralizes the feature extraction capability of our explicit stream (the local 1D-CNN pathway). Under these conditions, the relational logic is entirely implicit, forcing the model to rely almost exclusively on the implicit stream (Mamba) to capture the long-range association. However, because the DDI-int category suffers from extreme data scarcity in the training set, the state-space model struggles to converge on a robust semantic manifold for these intrinsically subtle logic patterns.

Therefore, the false negatives in this category perfectly illustrate the model’s boundary conditions: performance degrades when the explicit stream is starved of specific trigger words and the implicit stream is simultaneously starved of the training data required to map complex linguistic structures.

### Limitations and future work

Despite its robust performance, DuSSM faces challenges in extremely data-scarce classes, such as the DDI-int category. As noted in our error analysis, a critical vulnerability of the dual-stream architecture emerges when both streams are deprived of strong signals: the explicit stream lacks morphological triggers, and the implicit stream lacks sufficient training instances to map the subtle topological logic.

To mitigate this limitation and enhance the practical utility of the architecture in real-world clinical deployments, we recommend implementing a confidence-aware gating mechanism. When the reasoning module outputs a low-confidence probability distribution across all relation types for a sentence containing recognized biomedical entities, the system should default to a flag for review state. This human-in-the-loop approach ensures that highly ambiguous, trigger-less interactions are not dismissed as false negatives, thereby maintaining clinical safety.

Future work will explore several targeted architectural directions to overcome this dual-stream bottleneck. First, to address the data scarcity of underrepresented classes, we will investigate using general-purpose LLMs as offline synthetic data generators to augment the training manifold of categories like DDI-int. Second, to provide the implicit stream with stronger signals when lexical cues are absent, we plan to inject external topological priors—such as relational subgraphs from the UMLS or DrugBank knowledge bases—directly into the Mamba module’s latent state. This knowledge-guided state-space modeling would allow the system to infer relations based on biochemical priors even when the text sequence lacks explicit structural hints. Additionally, once the $𝒪(N^{2})$ contextual encoding bottleneck is fully bypassed by emerging linear foundational models, we aim to extend the DuSSM framework to document-level relation extraction, enabling the capture of complex interactions spanning across multiple paragraphs and complete electronic health records.

## Conclusion

In this study, we presented DuSSM, a parallel dual-stream state-space reasoning module designed to address the fundamental challenge of linguistic duality in biomedical relation extraction. By structurally disentangling explicit morphological patterns from implicit logical dependencies, DuSSM effectively mitigates the noise propagation and feature entanglement issues inherent in monolithic serial architectures. Our extensive evaluations across four heterogeneous benchmarks—DDI-2013, ChemProt, GAD, and EU-ADR—demonstrate that DuSSM consistently achieves robust performance across diverse biomedical and genomic domains. Crucially, the module’s exceptional precision on non-interaction cases (e.g., 94.32% on the ChemProt *None* class) directly addresses the critical clinical mandate of minimizing alert fatigue. Furthermore, although the overall pipeline is currently bottlenecked by the $𝒪(N^{2})$ contextual encoder, the empirical validation of the downstream reasoning module’s linear-time complexity (𝒪(N)), achieving inference latencies as low as 1.08 ms per sample, substantiates DuSSM’s immense potential for real-time, privacy-preserving deployment in resource-constrained health informatics environments.

Future research will focus on integrating targeted LLM prompting or external knowledge graphs to enhance few-shot performance on extremely rare interaction types. Moreover, as emerging linear foundational models gradually bypass the quadratic bottleneck of current contextual encoders, we aim to extend this dual-stream disentanglement paradigm to document-level extraction tasks. Ultimately, DuSSM provides a highly robust, efficient, and clinically transparent foundation for the next generation of automated biomedical decision support systems.
